# The GLP-1 receptor agonist exendin-4 reduces taurine and glycine in nucleus accumbens of male rats, an effect tentatively involving the nucleus tractus solitarius

**DOI:** 10.3389/fphar.2024.1439203

**Published:** 2024-08-16

**Authors:** Christian E. Edvardsson, Jesper Vestlund, Mia Ericson, Elisabet Jerlhag

**Affiliations:** ^1^ Department of Pharmacology, Institute of Neuroscience and Physiology, The Sahlgrenska Academy at the University of Gothenburg, Gothenburg, Sweden; ^2^ Department of Psychiatry and Neurochemistry, Institute of Neuroscience and Physiology, The Sahlgrenska Academy at the University of Gothenburg, Gothenburg, Sweden

**Keywords:** GLP-1, exenatide, microdialysis, dopamine, nucleus accumbens

## Abstract

The physiological effects of glucagon-like peptide-1 (GLP-1) are mainly centered on its ability to decrease blood glucose levels and facilitate satiety. Additional physiological functions have been identified by means of GLP-1 agonists such as exenatide (exendin-4; Ex4). In particular, Ex4 reduces the intake of natural and artificial rewards, effects that to some extent involve activation of GLP-1 receptors in the nucleus tractus solitarius (NTS). Although Ex4 acts in the brain, the neurochemical mechanisms underlying this activation are not fully elucidated. Investigating Ex4-induced neurochemical alterations in the nucleus accumbens (NAc) would be valuable for understanding its impact on reward-related behaviors. The aim of the present exploratory *in vivo* microdialysis study was therefore to study how Ex4, administered either systemically or locally into the NTS, influences classical neurotransmitters like dopamine, serotonin, noradrenaline, glutamate and GABA as well as additional players such as glycine, taurine and serine in NAc of male rats. We showed that Ex4 reduced extracellular levels of serine, taurine and glycine, where the latter two declines appear to involve activation of GLP-1R in the NTS. Besides, after systemic Ex4 injection the metabolites DOPAC, HVA, and 5HIAA are elevated. Where the increase in metabolites related to dopamine, but not serotonin, involves GLP-1 receptors in other areas than the NTS. Although the descriptive nature of the present data does not provide causality, it may however serve as an indication of mechanisms underlying how Ex4 may modulate reward-related behaviors.

## 1 Introduction

While a vast number of gut-brain peptides have been identified, glucagon-like peptide-1 (GLP-1) has gained recent attention. Although GLP-1 is primarily produced in the intestines [(for review see (Kanoski et al., 2016)], GLP-1-producing neurons have been identified in the nucleus tractus solitarius (NTS) of the brainstem (Merchenthaler et al., 1999). Intriguingly, these NTS neurons project to brain areas modulating reward, motivation, and intake of natural and artificial rewards, with the nucleus accumbens (NAc) being one such area (Merchenthaler et al., 1999; Alhadeff et al., 2012; Reiner et al., 2018). The physiological effects of GLP-1, which include decreasing blood glucose levels and promoting satiety [for review (Gribble and Reimann, 2021)], have led to the development of GLP-1 receptor (GLP-1R) agonists for managing type 2 diabetes and obesity [for review see (Andersen et al., 2018)]. One of these agonists is exenatide (exendin-4; Ex4), which has been used extensively in both preclinical and clinical studies. Additional physiological functions have been identified using Ex4, which has been found to reduce the intake of natural and artificial rewards [for review see (Eren-Yazicioglu et al., 2020; Jerlhag, 2023)]. Specifically, these effects are observed after both systemic and intra-NTS administration of Ex4 (Hayes et al., 2011; Kanoski et al., 2011; Dickson et al., 2012; Egecioglu et al., 2013c; Alhadeff and Grill, 2014; Richard et al., 2015; Bernosky-Smith et al., 2016; Vallof et al., 2019b; Vestlund and Jerlhag, 2020b).

Although Ex4 acts on GLP-1R in the brain (Goke et al., 1993), the underlying neurochemical mechanisms are not fully elucidated. NAc appears to be one central brain region through which Ex4 acts in the brain. Specifically, the rewarding properties of alcohol, alcohol consumption, feeding behaviors, and sexual interactions are attenuated after local infusion of Ex4 into the NAc (Dossat et al., 2011; Dickson et al., 2012; Dossat et al., 2013; Mietlicki-Baase et al., 2014; Abtahi et al., 2018; Vallof et al., 2019a; Vestlund and Jerlhag, 2020a; Colvin et al., 2020; Pierce-Messick and Pratt, 2020). Although the GLP-1Rs in NAc appear to modulate these reward-related behaviors, which are largely driven by an enhanced dopaminergic tone, the downstream neurochemical mechanisms influenced by Ex4 remain to be established. Considering that various afferents converge in the NAc, it is highly probable that Ex4 influences multiple neurotransmitters within the NAc. While reward-related behaviors are modulated by classical neurotransmitters like dopamine, serotonin, noradrenaline, glutamate, and GABA [for review (Klawonn and Malenka, 2018)], additional players such as glycine, taurine, and serine have been implied to be involved [for review see (Burgos et al., 2015; Soderpalm et al., 2017)]. Specifically, NAc glycine reduces alcohol intake (Molander et al., 2005; Olsson et al., 2021), taurine enhances alcohol-induced locomotor stimulation and reward memory (Valle et al., 2018; Ulenius et al., 2020) and serine prevents the sensitization induced by cocaine (D'Ascenzo et al., 2014). Given the importance of neurotransmission in NAc for reward-related behaviors, the overall aim of the present study was to simultaneously identify neurotransmitters in NAc affected by Ex4, specifically when administered at doses known to reduce motivated behaviors.

While the effects of Ex4 on central neurotransmission to a large extent remain unclear, previous studies have found that Ex4 does not alter dopamine in NAc (Egecioglu et al., 2013c; Vallof et al., 2019b), that Ex4 into the NTS increases dopamine turnover in NAc in male mice after a sexual encounter with a female (Vestlund and Jerlhag, 2020b) and that another GLP-1R agonist, semaglutide, enhances 3,4-Dihydroxyphenylacetic acid (DOPAC) and homovanillic acid (HVA) in NAc shell of male rats (Aranas et al., 2023). Moreover, Ex4 impacts serotonin and 5HIAA in other parts of the brain (Anderberg et al., 2016; Anderberg et al., 2017) and the GLP-1R agonist dulaglutide elevates *ex vivo* levels of noradrenaline in the prefrontal cortex (Vallof et al., 2020). Moreover, Ex4 enhances glutamatergic AMPA/Kainate signaling in NAc of outbreed rats (Mietlicki-Baase et al., 2014), suppresses the miniature excitatory postsynaptic currents of the dopaminergic projections from the VTA to NAc (Wang et al., 2015) and increases the frequency of spontaneous excitatory postsynaptic currents within the ventral tegmental area (VTA) (Mietlicki-Baase et al., 2013). While Ex4 enhances GABAergic neurotransmission in the dorsal striatum (Reiner et al., 2018), its impact on serine, glycine, and taurine has not been investigated. Given the scarce data on how Ex4, either systemically or into the NTS, influences transmission in the NAc, the aim of the present exploratory *in vivo* microdialysis study is to determine the effects of Ex4, at doses that suppress motivational behaviors, on classical neurotransmitters like dopamine, serotonin, noradrenaline, glutamate, and GABA as well as additional players such as glycine, taurine and serine in NAc of male rats. These simultaneous measurements will contribute to our understanding of Ex4’s underlying neurochemical mechanisms.

## 2 Material and methods

### 2.1 Animals

For the present *in vivo* microdialysis experiments, adult outbred male Wistar rats (250–300 g body weight; Charles River, Calco, Italy) were used, as Ex4 reduces alcohol and food intake in this strain (Dickson et al., 2012; Egecioglu et al., 2013c). The rats habituated to the animal facility for 1 week before initiation of any experimental procedures. Chow and water were freely available to the rats, which were housed in rooms with a temperature of 20 C and 50% humidity. They were housed in a regular 12 h light/dark cycle (light on at 7 a.m.), and the experiments were conducted during the animal’s light cycle (start at 8 a.m.) when they are inactive. The experiments were approved by Ethics Committee for Animal Experiments (ethical number: 1457/18 Gothenburg, Sweden).

### 2.2 Drugs

The GLP-1R agonist Ex4 (Tocris Bioscience, Abingdon, United Kingdom) was used as it activates central GLP-1R (Goke et al., 1993) without underlying mechanisms of action being identified. For intraperitoneal (IP) injections, a dose of 1.2 μg/kg, dissolved in vehicle (0.9% NaCl), was used as it previously has been shown to reduce alcohol and food intake (Dickson et al., 2012; Egecioglu et al., 2013c). For intra-NTS infusion, a dose of 0.05 µg in 0.5 µL, dissolved in vehicle (Ringer solution: NaCl 140 mM, CaCl_2_ 1.2 mM, KCl 3.0 mM and MgCl_2_ 1.0 mM; Merck KgaA, Darmstadt, Germany), was used as it previously has been found to decrease alcohol intake in rats (Vallof et al., 2019b) and sexual behaviors in mice (Vestlund and Jerlhag, 2020b). For both systemic and local administrations, an equal volume of vehicle was used (Egecioglu et al., 2013b; a;Egecioglu et al., 2013c; Vallof et al., 2019b).

### 2.3 *In vivo* microdialysis experiment

*In vivo* microdialysis is frequently used to measure the extracellular concentrations of transmitters, such as monoamines and amino acids, in a specific brain region [for review see (Darvesh et al., 2011)]. To confirm previous studies (Egecioglu et al., 2013c; Vallof et al., 2019b), we investigated if Ex4 by itself influences dopamine levels in the NAc. Given that noradrenaline, serotonin, glutamate, serine, glycine, and taurine in NAc modulate the intake of natural and artificial rewards, the effect of Ex4 thereof was studied. As another GLP-1R agonist, semaglutide, enhances 3,4-Dihydroxyphenylacetic acid (DOPAC) and homovanillic acid (HVA) in NAc shell of male rats (Aranas et al., 2023) we tested the hypothesis that Ex4 has a similar effect. Given that NTS is one region central for some but not all pharmacological effects of Ex4, Ex4 was administered either systemically or into NTS. It should be emphasized that all the above neurotransmitters were measured at precisely the same time in the rat.

A custom-made I-shaped microdialysis probe aiming at NAc shell was implanted 2 days prior to the microdialysis test as previously described (Edvardsson et al., 2021). The probe position was alternated between the right and left sides. Since the *in vivo* microdialysis method allows for monitoring extracellular transmitters in awake, freely moving animals (for review see (Darvesh et al., 2011)), it is useful to identify tentative mechanisms of action underlying Ex4 administration. To enable local infusion into the NTS, a guide (stainless steel, length 10 mm, with an o. d./i.d. of 0.6/0.45 mm) was implanted 1 mm below the surface of the skull. The coordinates for these areas were based on a rat brain atlas (Paxinos and Watson, 1998) as well as previous studies (Vallöf et al., 2019; Vestlund and Jerlhag, 2020b), and only rats with correct placements within all areas were included in the statistical analysis (Supplementary Figure S1A).

Two separate dialysis experiments, outlined in Supplementary Figure S2, were conducted in which Ex4 or vehicle was administered IP (experiment 1) or locally into the NTS (experiment two). The experiments were conducted in separate rats, and separate rats were used for placebo and Ex4 (n = 7-11 per treatment). On the day of the experiment, the dialysis probe was connected to a pump (U-864 Syringe Pump; Agnthos AB, Lidingö, Sweden) and perfused with Ringer solution at a rate of 2.0 μL/min. In experiment two, where the drug was infused locally into the NTS, a dummy cannula was after connection to the probe carefully inserted and retracted from the guide to remove clotted blood and hamper spreading depression. Following a 60-min habituation period, a dialysis sample was collected every 20 min throughout the entire experiment. The baseline of each neurotransmitter was defined as the average of the first three samples, and the % change in neurotransmitter levels (actual measurement/baseline*100), was calculated for each timepoint. Neither food nor water was available during these *in vivo* microdialysis experiments as both these parameters influence dopamine *per se*, and thus may interfere with the treatment effect. Neither was the body weight of the rat measured before and after the microdialysis experiments. Therefore, the effect of Ex4 on food intake and body weight cannot be determined.

In experiment 1, three samples (−40 min–0 min) were collected prior to Ex4 or vehicle injection (IP; at 0 min), and an additional nine samples (20–180 min) were collected. The second experiment followed a similar design, however Ex4 or vehicle was infused locally into the NTS (same side as the probe). In both experiments two separate treatment groups were included, and therefor rats were treated with either vehicle or Ex4.

As described before, a split fraction-system HPLC-EC system using chromatographic separation was used to detect the levels of noradrenaline, dopamine, serotonin, and their metabolites (DOPAC, HVA, 5-HIAA) in each sample (Waters et al., 2017) with the following modifications: The EC detector used was Ultimate ECD-3000RS, ThermoFisher Scientific, equipped with two amperometric cells, Ultimate 3000. No solenoid valve was used. A HPLC system using fluorescent detection (UltiMate 3000 HPLC system; Thermo Fisher Scientific, Gothenburg, Sweden) was used to separate and detect extracellular levels of amino acids (GABA, beta-alanine, glutamate, serine, glycine, and taurine) as described previously (Ulenius et al., 2020). Briefly, sodium azide was added to 5 µL dialysis sample for protection of the amino acids. Notably, GABA and beta-alanine were below the detection limit in these experiments. To identify the correct peaks in the chromatogram, external standards for all neurotransmitters and metabolites were used. Concentrations were determined using the Chromeleon7 software (Thermo Scientific Chromeleon Chromatography Data System).

### 2.4 Statistical analysis

The data were analyzed by a repeated two-way ANOVA, with Bonferroni *post hoc* test for comparisons between treatments, specifically at given time points. A probability value of *p* < 0.05 was considered statistically significant. Data are presented as mean ± SEM.

## 3 Results

### 3.1 Effects of systemic administration of Ex4 on monoaminergic neurotransmission in NAc

**FIGURE 1:**
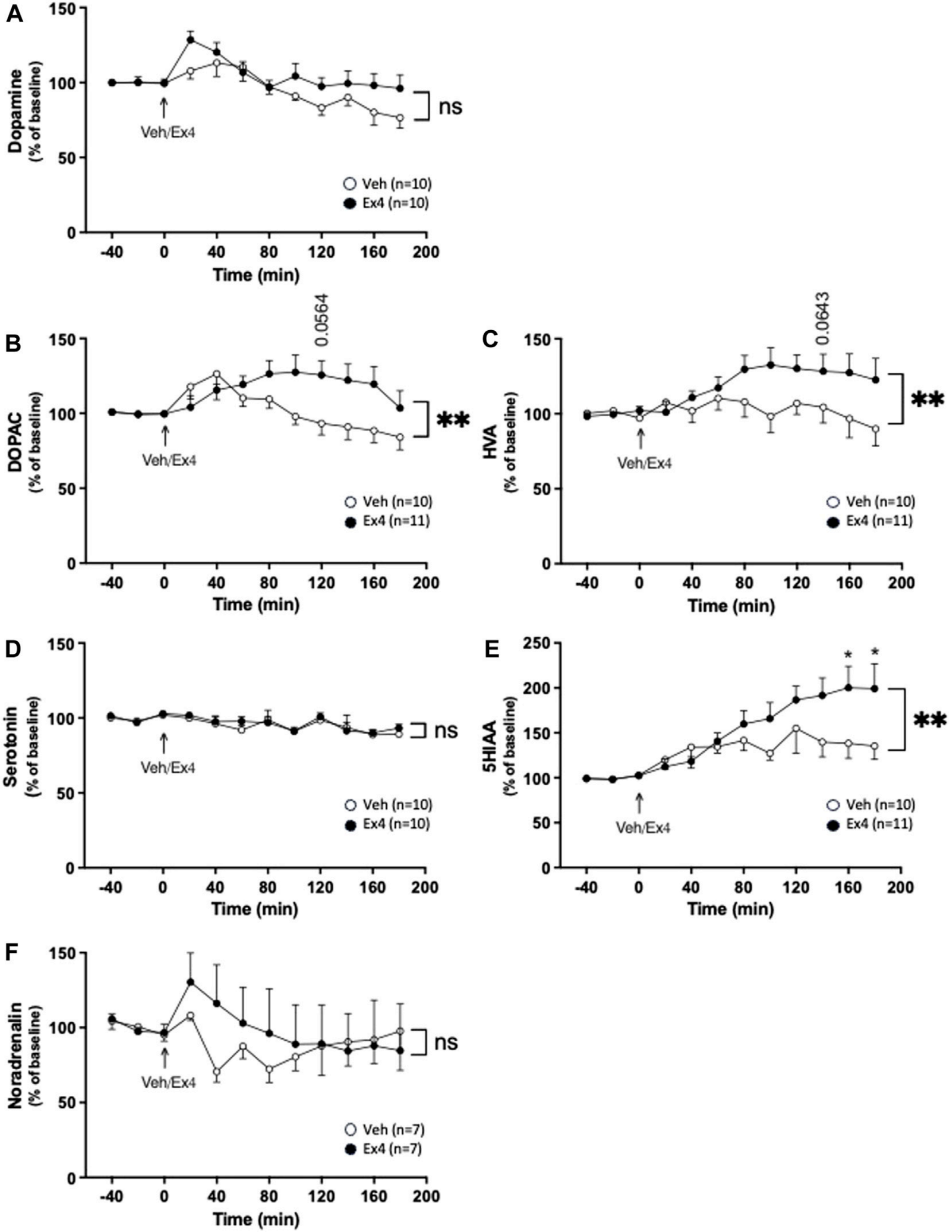
Effects of systemic exendin-4 on monoaminergic neurotransmission in nucleus accumbens shell. Compared to vehicle (Veh), intraperitoneal administration of exendin-4 (Ex4) **(A)** did not influence the extracellular dopamine levels, where it increased the extracellular levels of **(B)** dihydroxyphenylacetic (DOPAC), **(C)** homovanillic acid (HVA) in nucleus accumbens (NAc) shell. In contrast, Ex4 did not affect the **(D)** serotonin levels of NAc shell, whereas it elevated the extracellular levels of **(E)** 5-hydroxyindole acetic acid (5HIAA), an effect specifically evident at 160–180 min. **(F)** Moreover, noradrenalin was unaffected by Ex4. Data are analyzed with a repeated measure two-way ANOVA followed by Bonferroni post-hoc test and are presented as mean ± SEM. *p* > 0.05, no significance (ns), *p < 0.05, **p < 0.001.

As shown in previous studies, systemic administration of Ex4 did not influence the extracellular levels of dopamine in NAc shell as there was no overall treatment (F_1,18_ = 2.77, *p* = 0.1132) or interaction (F_11,198_ = 0.1.37, *p* = 0.1892; Figure 1A) effect. On the contrary, systemic Ex4 increased the extracellular levels of DOPAC as there tended to be an overall treatment (F_1,19_ = 4.02, *p* = 0.0593), as well as interaction (F_11,209_ = 2.85, *p* = 0.0017; Figure 1A, B) effect. This increase tended to be evident at 140 min (*p* = 0.0564) after Ex4 administration. On a similar note, systemic Ex4 elevated HVA in NAc since there was an overall treatment (F_1,19_ = 3.28, *p* = 0.0860), and interaction (F_11,209_ = 2.40, *p* = 0.0079; Figure 1C, B) effect. Specifically, the HVA levels tended to be higher at 120 min (*p* = 0.0643) after Ex4. When it comes to serotonin, there was no overall treatment (F_1,18_ = 0.22, *p* = 0.6429), or interaction F_11,198_ = 0.28, *p* = 0.9894; Figure 1D) effect, indicating that Ex4 did not alter serotonin in NAc. In contrast, Ex4 increased the extracellular levels of 5HIAA as there tended to be an overall treatment effect (F_1,19_ = 3.17, *p* = 0.0909), and was a significant interaction effect (F_11,209_ = 2.43, *p* = 0.0072; Figure 1E). Ex4 increased 5HIAA 160–180 min (*p* < 0.05) after treatment. Systemic Ex4 did not change noradrenaline as there was no overall treatment (F_1,12_ = 0.26, *p* = 0.6995) or interaction (F_11,132_ = 0.80, *p* = 0.6355; Figure 1F) effect.

### 3.2 Effects of intra-NTS administration of Ex4 on monoaminergic neurotransmission in NAc

**FIGURE 2:**
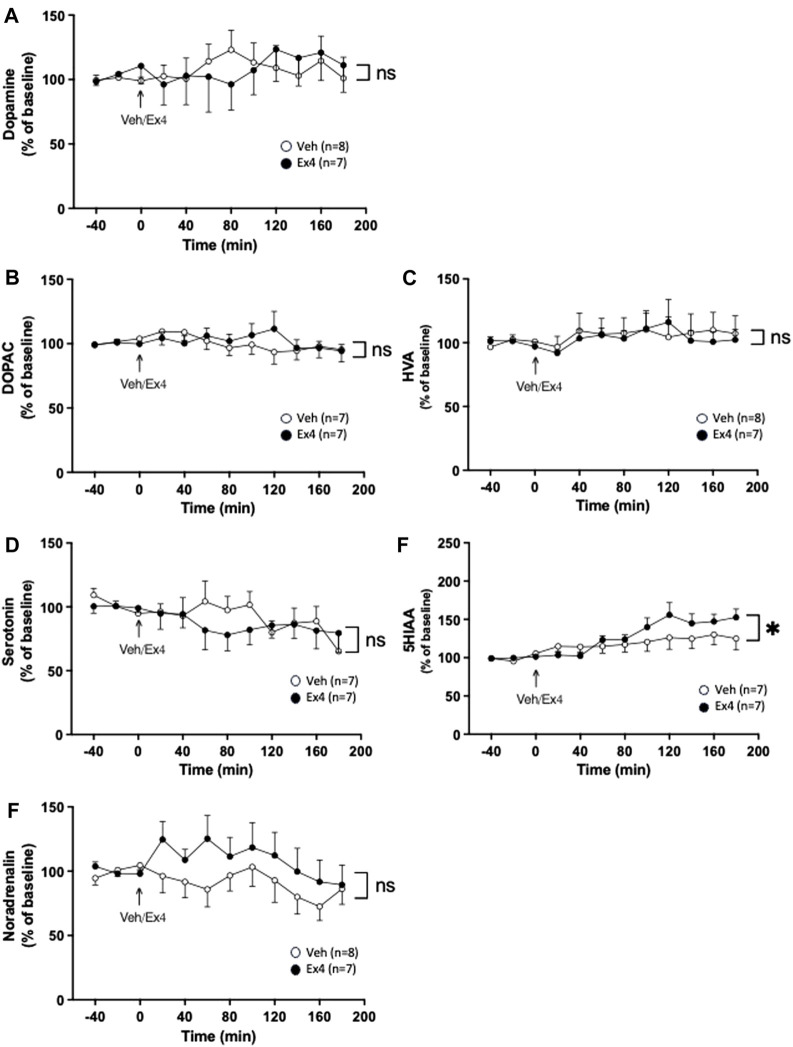
Effects of infusion of exendin-4 into nucleus tractus solitarius on monoaminergic neurotransmission in nucleus accumbens shell. The extracellular levels of **(A)** dopamine, **(B)** dihydroxyphenylacetic (DOPAC), **(C)** homovanillic acid (HVA) and **(D)** serotonin in nucleus accumbens (NAc) were unchanged after infusion of exendin-4 (Ex4) into the nucleus tractus solitarius (NTS). **(E)** Compared to vehicle (Veh), Ex4 into NTS caused an elevation of 5-hydroxyindole acetic acid (5HIAA) levels in NAc shell. **(F)** Whereas noradrenalin was similar between treatment groups. Data are analyzed with a repeated measure two-way ANOVA followed by Bonferroni post-hoc test and are presented as mean ± SEM. *p* > 0.05, no significance (ns), *p < 0.05.

Intra-NTS infusion of Ex4 did not change the extracellular levels of dopamine as there was no overall treatment (F_1,13_ = 0.00, *p* = 0.9668) or interaction (F_11,143_ = 0.61, *p* = 0.8155; Figure 2A) effect. Similarly, there was no treatment (F_1,12_ = 0.04, *p* = 0.8519) or interaction (F_11,132_ = 1.07, *p* = 0.3888; Figure 2B) effect on the extracellular levels of DOPAC in NAc. Neither was there a treatment (F_1,13_ = 0.03, *p* = 0.8615) or interaction (F_11,143_ = 0.30, *p* = 0.9850; Figure 2C) effect on HVA. Ex4 into NTS did not alter the extracellular levels of serotonin since there was no treatment (F_1,12_ = 0.19, *p* = 0.6738) or interaction (F_11,132_ = 0.97, *p* = 0.4739; Figure 2D) effect. After intra-NTS infusion of Ex4, there was an overall interaction (F_11,132_ = 2.38, *p* = 0.0103; Figure 2E), but no treatment (F_1,12_ = 0.76, *p* = 0.4007) effect on the 5HIAA levels in NAc. Contrarily, there was no overall treatment (F_1,13_ = 1.19, *p* = 0.2948) or interaction (F_11,143_ = 0.98, *p* = 0.4664; Figure 2F) effect on the extracellular levels of noradrenaline in NAc.

### 3.3 Effects of systemic administration of Ex4 on amino acids in NAc

**FIGURE 3:**
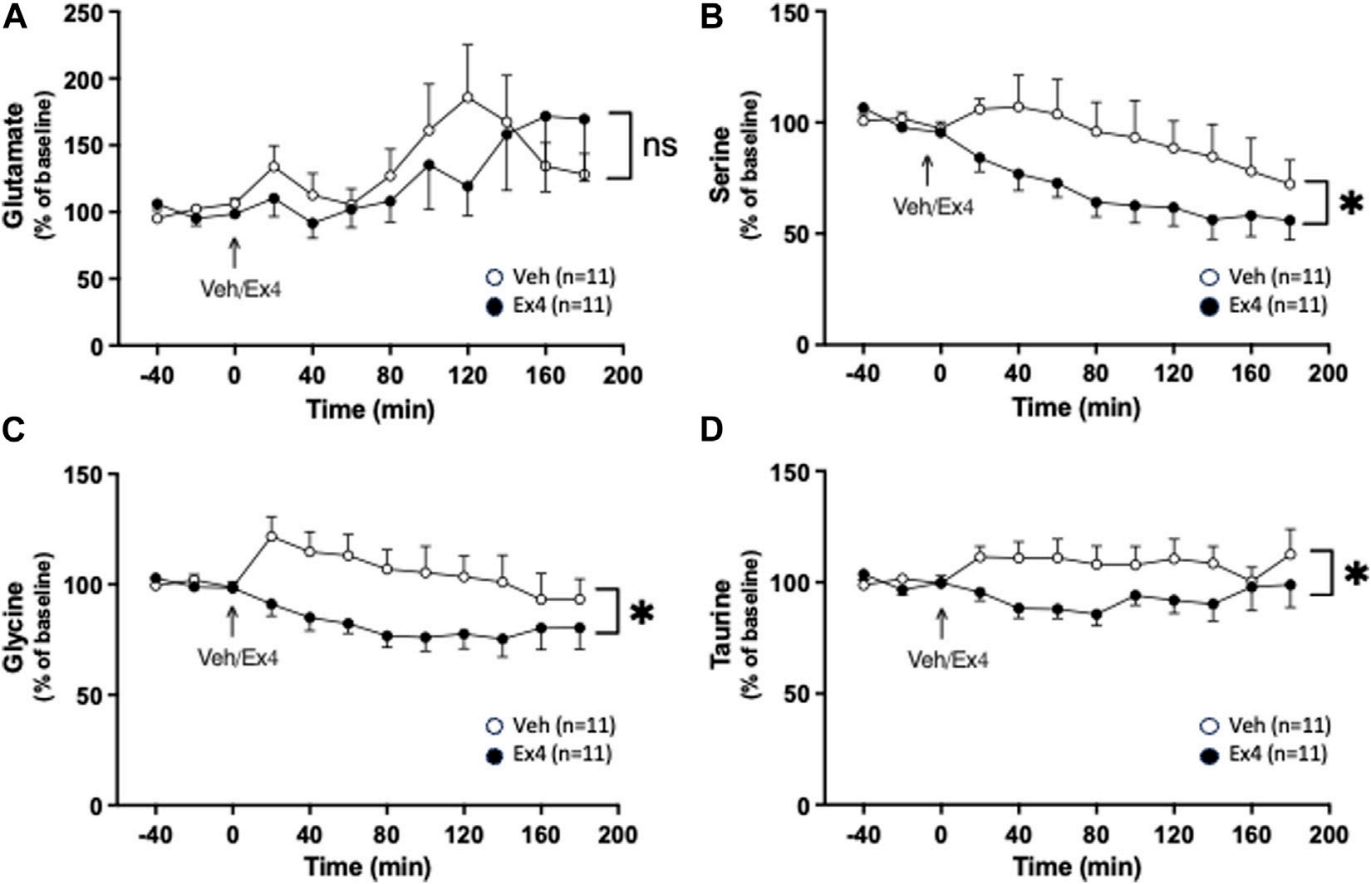
Effects of systemic exendin-4 on amino acids in nucleus accumbens shell. **(A)** The extracellular levels of glutamate were similar between rats treated with exendin-4 (Ex4) and vehicle (Veh), whereas Ex4 reduced the extracellular levels of **(B)** serine, **(C)** glycine, and **(D)** taurine. Data are analyzed with a repeated measure two-way ANOVA followed by Bonferroni post-hoc test and are presented as mean ± SEM. *p* > 0.05, no significance (ns), *p < 0.05, **p < 0.001.

After systemic administration of Ex4 there was no treatment (F_1,20_ = 0.11, *p* = 0.7464) or interaction (F_11,220_ = 1.11, *p* = 0.3517; Figure 3A) effect on the extracellular levels of glutamate. In contrast, systemic Ex4 reduced the extracellular levels of serine since there tended to be an overall treatment (F_1,20_ = 3.59, *p* = 0.0725) and significant interaction (F_11,220_ = 2.08, *p* = 0.0231; Figure 3B) effect. On a similar note, glycine was lowered by systemic Ex4 as there was an overall treatment (F_1,20_ = 5.20, *p* = 0.0337) and interaction (F_11,220_ = 2.11, *p* = 0.0210; Figure 3C) effect. Specifically, Ex4 lowered glycine 80 min (*p* < 0.05) after treatment. Moreover, there tended to be an overall treatment (F_1,20_ = 3.84, *p* = 0.0640) and significant interaction (F_11,220_ = 2.51, *p* = 0.0368; Figure 3D) effect on taurine in NAc, indicating that treatment reduces this amino acid.

### 3.4 Effects of intra-NTS administration of Ex4 on amino acids in NAc

**FIGURE 4:**
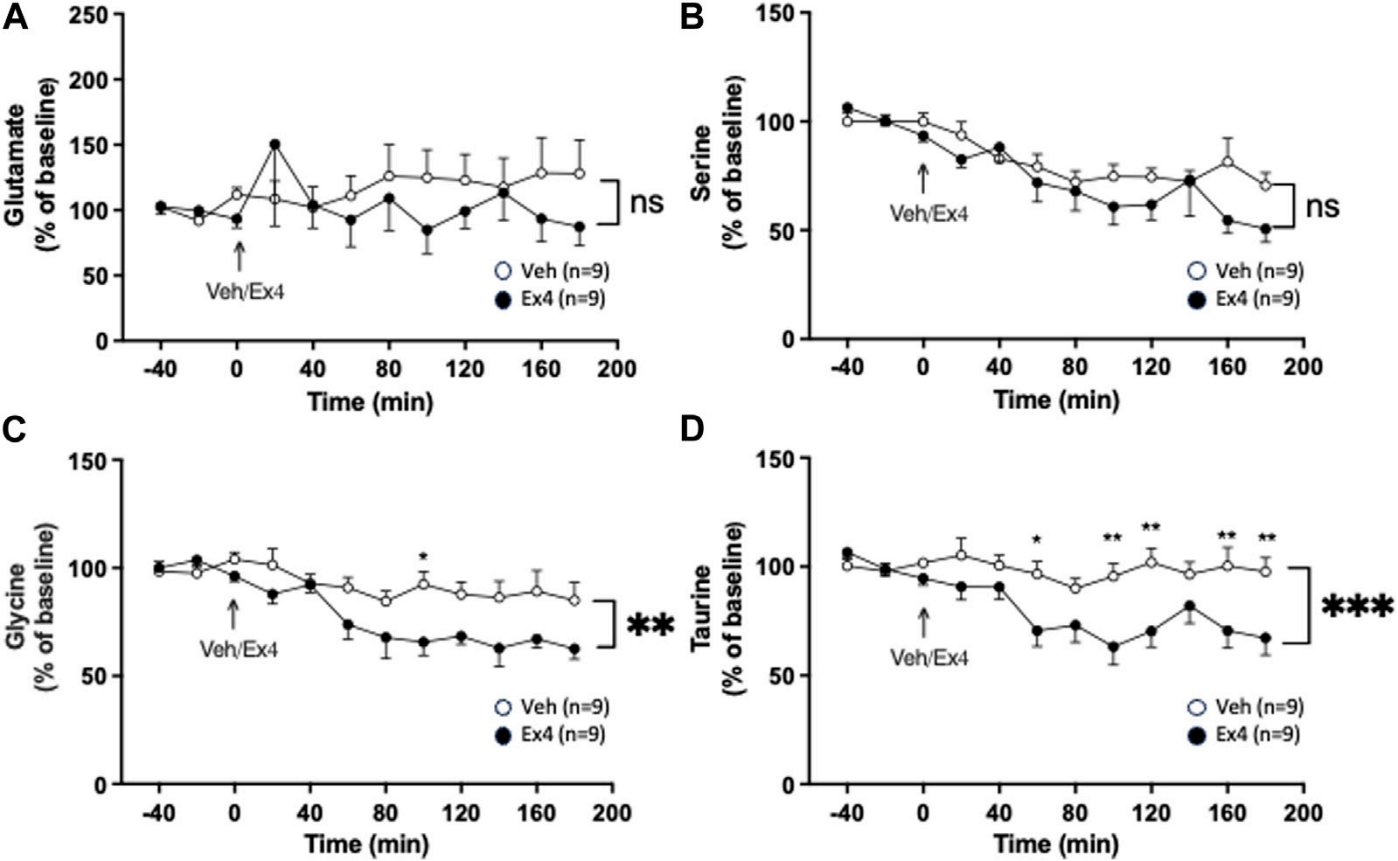
Effects of infusion of exendin-4 into nucleus tractus solitarius on amino acids in nucleus accumbens shell. The extracellular levels of **(A)** glutamate and **(B)** serine were similar in nucleus accumbens (NAc) shell of rats after infusion of exendin-4 (Ex4) or vehicle (Veh) into the nucleus tractus solitarius (NTS). Contrarily, Ex4 into NTS reduced the extracellular levels of both **(C)** glycine and **(D)** taurine in NAc shell. Posthoc analysis revealed that Ex4 reduced glycine at 100 min (*p* < 0.05) and taurine at 60 (*p* < 0.05), 100–120 (*p* < 0.01), 160–180 (*p* < 0.01) minutes. Data are analyzed with a repeated measure two-way ANOVA followed by Bonferroni post-hoc test and are presented as mean ± SEM. *p* > 0.05, no significance (ns), *p < 0.05, ** *p* > 0.001.

Intra-NTS administration of Ex4 did not have an overall treatment (F_1,16_ = 0.38, *p* = 0.5475) or interaction (F_11,176_ = 0.98, *p* = 0.4655; Figure 4A) effect on the extracellular levels of glutamate. Neither was there an overall treatment (F_1,16_ = 1.45, *p* = 0.2465) effect, but tended to be an overall interaction (F_11,176_ = 1.78, *p* = 0.0604; Figure 4B) effect on serine in NAc shell. On the other hand, intra-NTS Ex4 reduced the extracellular levels of glycine as there was an overall treatment (F_1,16_ = 8.21, *p* = 0.0112) and interaction (F_11,176_ = 2.46, *p* = 0.0070; Figure 4C) effect. The lowered glycine levels were found 100 min (*p* < 0.05) after Ex4 infusion into NTS. Similarly, Ex4 into NTS decreased taurine as there was an overall treatment (F_1,16_ = 11.58, *p* = 0.0036) and interaction F_11,176_ = 3.15, *p* = 0.0007; Figure 4D) effect. The taurine levels were decreased 60 (*p* < 0.05), 100–120 (*p* < 0.01), 160–180 (*p* < 0.01) minutes after NTS-Ex4.

## 4 Discussion

The present descriptive *in vivo* microdialysis study revealed that systemic administration of Ex4 did not affect the extracellular levels of dopamine, serotonin, and noradrenaline in NAc, whereas it increased the metabolites DOPAC, HVA, and 5HIAA (Table 1). As further evident in these rats, systemic Ex4 concurrently reduced serine, glycine, and taurine without altering glutamate in NAc (Table 1). Additionally, the involvement of GLP-1R in the NTS appeared to influence some of these observed effects. Specifically, intra-NTS administration of Ex4 elevated 5HIAA without influencing any of the other monoamines or their metabolites in NAc (Table 1). As further measured simultaneously in these rats, Ex4 into NTS did not interact with glutamate or serine, whereas it reduced both glycine and taurine (Table 1).

**TABLE 1:** Summary of obtained effects.

		Systematic administration	Intra-NTS administration
Monoaminergic neurotransmission	Dopamine	↮	↮
	DOPAC	↑	↮
	HVA	↑	↮
	Serotonin	↮	↮
	5HIAA	↑	↑
	Noradrenaline	↮	↮
Amino acids	Glutamate	↮	↮
	Serine	↓	↮
	Glycine	↓	↓
	Taurine	↓	↓

Summary of effects of systemic or intra-NTS, administration of Ex4 on monoaminergic neurotransmission and amino acids in nucleus accumbens shell of male rats. All neurotransmitters are measured at precisely the same time in the rat. ↓ decrease, ↑ increase, and ↮ no treatment effect.

The reduced extracellular levels of serine after systemic, but not intra-NTS, injection of Ex4 may be due to enhanced transportation of serine into astrocytes or an ablated release from neurons or astrocytes (Martineau et al., 2013). In this context, the lowered serine levels may be caused by a decreased glutamatergic tone (Mothet et al., 2000). Supportively, studies of NAc slices from rats reveal that Ex4 decreased the evoked population spike amplitude as an indication of reduced glutamatergic tone (Vestlund et al., 2020). On the other hand, Ex4 did not alter the extracellular levels of glutamate in the present study and Ex4 enhanced glutamatergic AMPA/Kainate signaling in NAc in another study (Mietlicki-Baase et al., 2014). However, the plausibility that Ex4 changes synaptic glutamate in NAc should be considered as the present neurochemical method cannot determine such detailed effects. While the downstream effect of reduced serine levels should be defined in future studies, it may cause a decrease in NMDA receptor activity since serine binds to the glycine site on the NR1 subunit of NMDA receptors (Mothet et al., 2000). It should also be noted that reduced extracellular levels of serine after Ex4, an agent known to suppress drug-related behaviors, is opposite to other studies on serine and reward-related behaviors. Specifically, enhanced serine levels in NAc suppress the ability of cocaine to cause locomotor sensitization, condition a place preference, and cocaine reinstatement (D'Ascenzo et al., 2014). Furthermore, elevated serine decreases aversion-resistant alcohol drinking in rodents (Seif et al., 2015). However, it is well known that neuroadaptive changes linked to addictive drugs are complex, and diverging molecular changes can occur at the same time at different levels in NAc. Adding to the complexity is the fact that different effects can be observed depending on the drug-exposure protocols.

The present *in vivo* microdialysis study further showed that Ex4, both after its systemic and intra-NTS injection, reduced the extracellular levels of glycine. As glycine has been found to elevate dopamine in NAc (Molander et al., 2005; Olsson et al., 2021), we suggest that decreased glycine levels disrupt the sensitivity of the mesolimbic dopamine system and thereby rendering it less responsive to reward activation. Supportively, glycine receptor activation reduces the GABAergic input to the VTA (Zheng and Johnson, 2001), which in turn enhances the release of dopamine in NAc (Lof et al., 2007). It should however be noted that the obtained data are contradictory to the ability of glycine, systemic or into NAc, to reduce alcohol drinking in rats (Molander et al., 2005; Chau et al., 2010). This discrepancy may lie in the ability of Ex4 to block, whereas glycine augments (Lido et al., 2011), the alcohol-induced dopamine release in NAc (Egecioglu et al., 2013c). It is thus possible that glycine levels in opposite directions may yield a comparable outcome depending on the activation of glycine receptors expressed on either D1-like or D2-like medium spiny neurons - two circuits known to exert contrasting influences on the activity of the mesolimbic dopamine system.

We further found that Ex4 decreased the extracellular levels of taurine, a well-known glycine receptor agonist. The findings that taurine augments the dopamine levels in NAc (Ericson et al., 2006) and that alcohol elevates accumbal taurine (De Witte et al., 1994; Dahchour et al., 1996; Quertemont et al., 2000), led us to hypothesize that Ex4 may prevent activation of the mesolimbic dopamine system *via* reduced taurine levels. Supportively, repeated taurine administration enhances alcohol-induced locomotor stimulation (Ulenius et al., 2020), and elevated taurine levels are required for alcohol to increase dopamine levels in NAc (Ericson et al., 2013). On a similar note, taurine increases attention and reward memory in rats (Valle et al., 2018).

We further found that activation of GLP-1R in NTS appears to be important for the ability of Ex4 to lower both taurine and glycine, but not serine. It however remains to be determined if the NTS-NAc projection co-releases these amino acids or if this projection influences the release of these amino acids directly in the NAc. Within the NTS, Ex4 has been suggested to control reward-related behaviors (Alhadeff et al., 2012; Richard et al., 2015; Vallöf et al., 2019; Vestlund and Jerlhag, 2020b) *via* action on presynaptic GLP-1R (Vrang et al., 2007; Hayes et al., 2009; Reiner et al., 2016), rather than post-synaptic GLP-1R (Hisadome et al., 2010). Yet, this potential causality remains to be determined in upcoming studies.

Following systemic injection of Ex4, enhanced extracellular levels of the dopamine metabolites DOPAC and HVA were observed in NAc shell. On a similar note, another GLP-1R agonist, semaglutide, increased the accumbal levels of DOPAC and HVA in this area when alcohol was onboard (Aranas et al., 2023). Furthermore, Ex4 increased the dopamine turnover in NAc in male mice exposed to sex (Vestlund and Jerlhag, 2020b). As this increase was not demonstrated after Ex4 infusion into NTS, other brain areas may mediate this effect. While the ability of semaglutide together with alcohol to increase DOPAC and HVA involved elevated expression of enzymes metabolizing dopamine in NAc (Aranas et al., 2023), this expression effect remains to be determined for Ex4. Another metabolite influenced by Ex4 was 5HIAA, where increased extracellular 5HIAA levels were observed in NAc shell after both systemic and intra-NTS Ex4 administration. An effect of Ex4 on 5HIAA is to some extent supported by the *ex vivo* neurochemical data from mice exposed to sex, where Ex4 tended to increase 5HIAA in NAc after its administration into NTS (Vestlund and Jerlhag, 2020b). The findings that semaglutide enhanced the expression of the enzyme MAO (Aranas et al., 2023), which converts serotonin to 5HIAA, raises the possibility that Ex4 *via* such mechanisms causes elevated levels of 5-HIAA in NAc. Support for an interaction between serotonin and GLP-1R has been provided before as the GLP-1-producing neurons of the NTS are activated by serotonin (Holt et al., 2017). It should be noted that the Ex4-induced increase in 5HIAA is greater after systemic compared to NTS infusion, indicating that activation of GLP-1R in additional areas may be responsible for this effect. One of these could be dorsal raphe, known to project to NAc, and where Ex4 acts to reduce feeding (Anderberg et al., 2017). Support for a general increase in serotonergic metabolism in the brain after Ex4 treatment is provided as central Ex4 increases serotonin turnover in the amygdala and hypothalamus (Anderberg et al., 2016; Anderberg et al., 2017). Although Ex4 increased 5-HIAA in NAc, no evident treatment effect on 5 H T was observed. Supportively, the extracellular levels of serotonin were unaltered by an intra-NTS infusion of Ex4 in rats (Vallof et al., 2019b). This increased rate of serotonin synthesis could be a tentative compensatory mechanism; however, this remains to be studied.

Strengths associated with the present descriptive microdialysis study are that all neurotransmitters were measured simultaneously in each rat and that separate rats were used for systemic/NTS and Ex4/placebo injections. The present *in vivo* microdialysis study is also associated with various limitations, one being measurements of extracellular instead of synaptic neurotransmitter levels, calling for further research to elucidate detailed effects in NAc. Additionally, the absence of GABA and beta-alanine detection in the current samples is a limitation as Ex4 has been shown to act *via* GABAergic signaling (Reiner et al., 2018; Fortin et al., 2020). Moreover, the present study is limited to male subjects, and additional studies in females are warranted. Given the mechanisms through which Ex4 changes neurotransmission in NAc cannot be determined through the present experiments, upcoming studies should be designed to establish this in detail. As the present study is descriptive, the physiological importance of the observed changes in extracellular concentrations remains to be determined in upcoming tests. We however hypothesize that Ex4 through these changes in neurotransmission may prevent activation of the mesolimbic system and thereby suppress reward-related behaviors. Supportively, Ex4 has previously been shown to attenuate reward-related behaviors associated with alcohol, addictive drugs, and natural rewards which all are driven *via* the enhanced activity of the mesolimbic system (for review see (Jerlhag, 2023)). Specifically, these suppressive effects of Ex4 are observed after both systemic and intra-NTS administration of Ex4 (Hayes et al., 2011; Kanoski et al., 2011; Dickson et al., 2012; Egecioglu et al., 2013c; Alhadeff and Grill, 2014; Richard et al., 2015; Bernosky-Smith et al., 2016; Vallof et al., 2019b; Vestlund and Jerlhag, 2020b). As shown before, Ex4 prevented such effects without suppressing the levels of dopamine in NAc below baseline (for revies see (Jerlhag, 2023)), indicating that the use of Ex4 is not associated with anhedonia and sedation. Supportively, the present study reveals that Ex4 did not alter the dopamine levels in NAc after its systemic or intra-NTS administration. Neither did Ex4 influence the noradrenergic neurotransmission in NAc.

In summary, this study reveals decreased extracellular levels of serine, taurine, and glycine following Ex4 administration, where the latter two potentially involve GLP-1R activation in the NTS. As shown before, Ex4 enhances the metabolism of dopamine resulting in elevated levels of DOPAC and HVA, most likely *via* other regions than NTS. Although the descriptive nature of the present data does not provide causality, it may however serve as an indication of mechanisms through which Ex4 modulates the activity of the mesolimbic dopamine system and reward-related behaviors.

## Data Availability

The raw data supporting the conclusion of this article will be made available by the authors, without undue reservation.
