# Supplementary material for: The GLP-1 receptor agonist exendin-4 reduces taurine and glycine in nucleus accumbens of male rats, an effect tentatively involving the nucleus tractus solitarius
Source: Front Pharmacol. 2024 Aug 16;15:1439203. doi: 10.3389/fphar.2024.1439203 (PMC11362053; doi:10.3389/fphar.2024.1439203)
Supplement: Supplementary file 1 [file Presentation1.PPTX]

## Slide 1
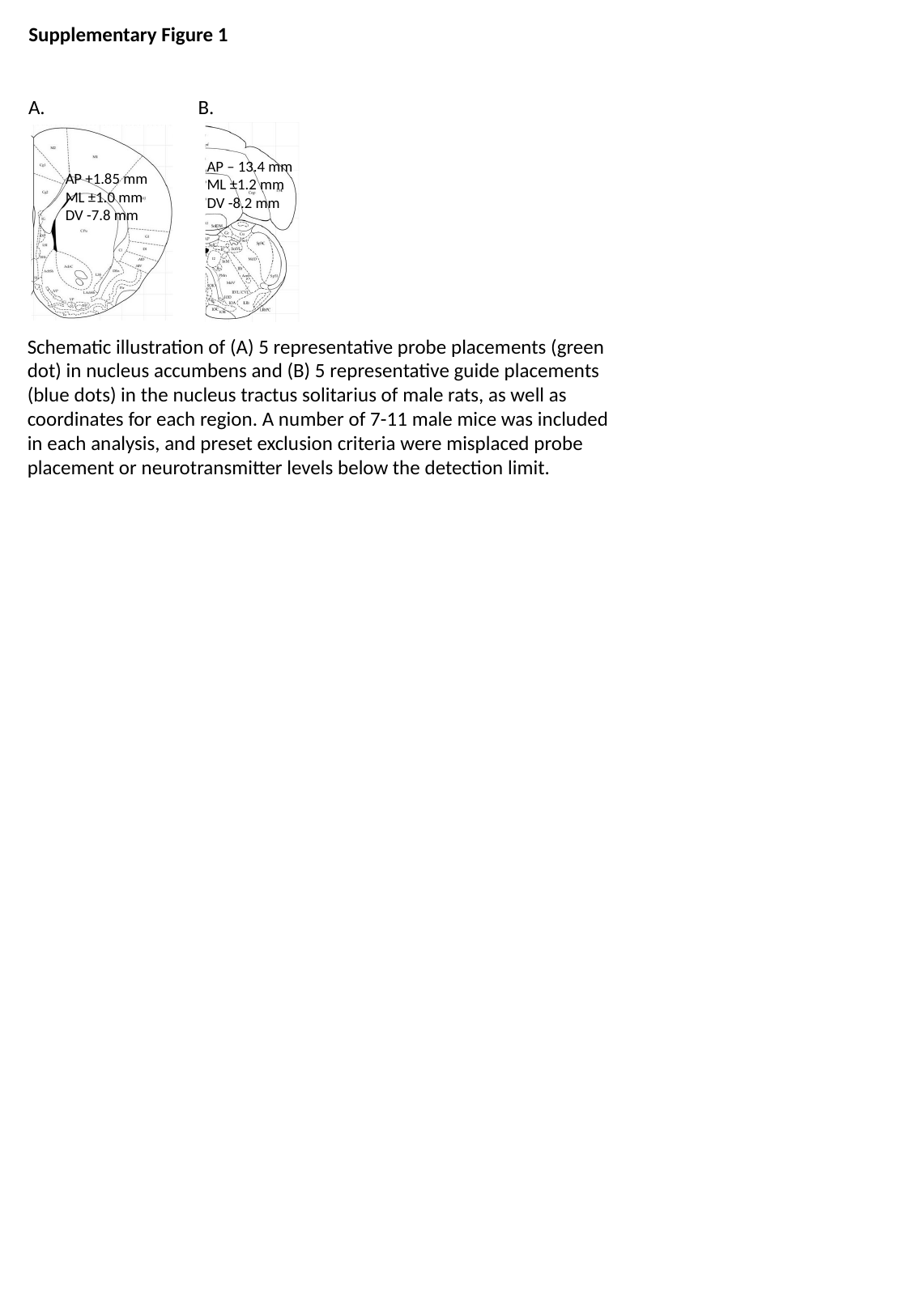

Supplementary Figure 1
A. B.
AP – 13.4 mm
ML ±1.2 mm
DV -8.2 mm
AP +1.85 mm
ML ±1.0 mm
DV -7.8 mm
Schematic illustration of (A) 5 representative probe placements (green dot) in nucleus accumbens and (B) 5 representative guide placements (blue dots) in the nucleus tractus solitarius of male rats, as well as coordinates for each region. A number of 7-11 male mice was included in each analysis, and preset exclusion criteria were misplaced probe placement or neurotransmitter levels below the detection limit.

## Slide 2
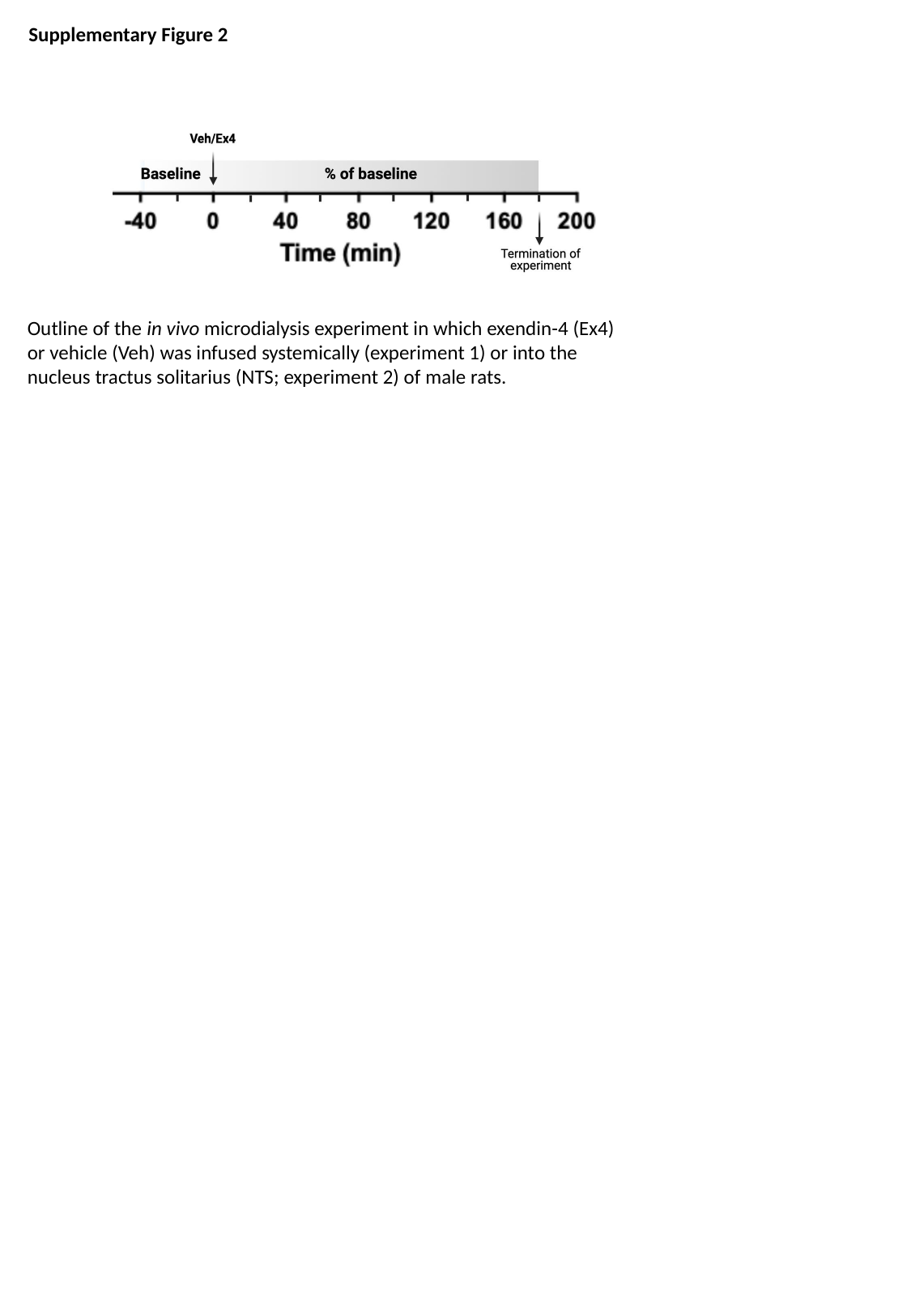

Supplementary Figure 2
Outline of the in vivo microdialysis experiment in which exendin-4 (Ex4) or vehicle (Veh) was infused systemically (experiment 1) or into the nucleus tractus solitarius (NTS; experiment 2) of male rats.
